# The impact of the first three months of the COVID-19 pandemic on the Australian trans community

**DOI:** 10.1080/26895269.2021.1890659

**Published:** 2021-03-11

**Authors:** Sav Zwickl, Lachlan M. Angus, Alex Wong Fang Qi, Ariel Ginger, Kalen Eshin, Teddy Cook, Shalem Y. Leemaqz, Eden Dowers, Jeffrey D. Zajac, Ada S. Cheung

**Affiliations:** a Trans Health Research Group, Department of Medicine (Austin Health), The University of Melbourne, Heidelberg, Victoria, Australia; b Department of Endocrinology, Austin Health, Gender Clinic, Heidelberg, Victoria, Australia; c La Trobe University, Bundoora, Victoria, Australia; d ACON Health, Surry Hills, New South Wales, Australia; e Flinders University, Adelaide, South Australia, Australia; f Swinburne University of Technology, Hawthorn, Victoria, Australia

**Keywords:** Coronavirus, COVID-19, depression, suicidality, transgender

## Abstract

**Background:**

Trans and gender diverse individuals (people who identify with a gender different to what was presumed for them at birth) are one of the most medically and socially marginalized groups in our community. The COVID-19 pandemic may compound preexisting depression and thoughts of self-harm or suicide.

**Aim:**

We aimed to explore the impact of the COVID-19 pandemic on the Australian trans community.

**Methods:**

An online cross-sectional survey was conducted between 1st May 2020 and 30th June 2020, amidst strict Australia-wide social restrictions. Australian trans people aged ≥16 years were eligible to participate. Survey questions explored the impact of the COVID-19 pandemic on living situation, employment, financial situation, and healthcare. Logistic regression to assess negative impacts due to COVID-19 on depression and thoughts of self-harm or suicide (measured by Patient Health Questionnaire-9 (PHQ-9) are presented as odds ratios (95% confidence interval)).

**Results:**

Of 1019 participants, 49.6% reported experiencing financial strain, 22% had reduced working hours, and 22.4% were unemployed (three times the national rate). Concerningly, 61.1% experienced clinically significant symptoms of depression (Patient Health Questionnaire-9 score ≥10), considerably higher than pre-COVID rates for the trans community and over twice the national rate. Moreover, 49% reported thoughts of self-harm or suicide (over three times the national rate) which was more likely if a person experienced cancelation or postponement of gender-affirming surgery (OR 1.56 (1.04, 2.35)), financial strain (OR 1.80 (1.36, 2.38)), or felt unsafe or afraid in their household (OR 1.96 (1.23, 3.08)).

**Discussion:**

Given rates of clinically significant depression and thoughts of self-harm or suicide are far higher in trans people than the general population, specific strategies to improve mental health in the trans community during the COVID-19 pandemic must be made a priority for policymakers, researchers, and health service providers to prevent suicide.

Supplemental data for this article is available online at https://doi.org/10.1080/26895269.2021.1890659

## Introduction

Transgender and gender diverse (referred to herein as *trans*) refers to people who have a gender that is different to what was presumed for them at birth and includes binary (male or female) and non-binary gender identities. Trans individuals comprise an estimated 0.5–4.5% of the adult population (Åhs et al., 2018; Crissman et al., 2017; Lai et al., 2010) but face numerous health disparities and are one of the most medically and socially marginalized groups in our community (Bockting et al., 2013; Bretherton et al., 2021).

Prior to the COVID-19 pandemic, trans people in Australia faced high rates of discrimination, sexual assault, physical and verbal abuse, homelessness, and multiple barriers to healthcare access (Bretherton et al., 2021; Strauss et al., 2020). Few Australian studies have used a validated diagnostic measure to estimate the rate of depression and thoughts of self-harm or suicide in the trans community. Pitts et al. (2009) reported 36.2% of trans adults in Australia met the criteria for a current major depressive episode and 25% reported thoughts of self-harm or suicide in the prior two weeks, as assessed by the Primary Care Evaluation of Mental Disorders Patient Questionnaire. More recently, Hyde et al. (2013) reported that 43.7% of trans adults experienced clinically significant depression and 53.6% reported thoughts of self-harm or suicide in the preceding two weeks, as assessed by the Patient Health Questionnaire-9 (PHQ-9). Comparatively, only 3.7% of a random sample of the Australian population in 2015 met criteria for clinically significant depression based on the PHQ-9 (Kiely & Butterworth, 2015). Over 40% of trans adults (Bretherton et al., 2021) and young people (Strauss et al., 2020) have attempted suicide, and deaths by suicide in trans people have been reported to be significantly higher than the general population (Wiepjes et al., 2020). Similar high rates of depression and suicidality in trans communities have been reported internationally (Adams et al., 2017; Bockting et al., 2013).

As many as one in five have experienced discrimination from healthcare providers and consequently, many trans people struggled to access health services even before the COVID-19 pandemic (Bretherton et al., 2021; Jaffee et al., 2016). Social disadvantage is likely to increase the risk of illness and mortality during the COVID-19 pandemic, increasing fear and anxiety experienced by marginalized groups.

Recent research has also begun to explore resilience and protective factors against depression, suicidality, and other mental health comorbidities in the trans community. For example, access to gender-affirming hormones and surgery (Riggs et al., 2014; White Hughto & Reisner, 2016) and social support and connection with the trans community (Moody & Smith, 2013; Sherman et al., 2020) have been shown to improve mental health and improve quality of life.

In Australia, the early months of the COVID-19 pandemic were characterized by relatively low positive cases and deaths due to strict social restrictions. International borders were closed and non-essential travel within and between states and territories was limited. Schools and universities transitioned to home-based online learning, and employees were instructed to work from home where possible. Many allied health services, including psychology and non-essential businesses were closed or changed to an online model for service delivery (i.e., telehealth). All elective surgery, including gender-affirming surgery was canceled or postponed to conserve healthcare resources. Rates of domestic violence were reported to have exponentially increased during extended periods of “lockdown” restrictions worldwide (Bradbury-Jones & Isham, 2020). With government orders to stay at home, trans people were potentially isolated with family or household members who may not have accepted their gender identity.

The COVID-19 pandemic has brought significant psychological distress on a global scale to many populations (Fisher et al., 2020; Pierce et al., 2020). Suicidal behavior is likely to be present for longer and peak later than the pandemic (Gunnell et al., 2020), and concern for the trans community has been raised as a priority (Wang et al., 2020). As such, we aimed to explore and understand the impact of the COVID-19 pandemic on the living situation, employment, financial status, depression, and thoughts of self-harm or suicide of the trans community in Australia. We hypothesized that the trans community would experience higher rates depression and thoughts of self-harm or suicide during the COVID-19 pandemic, as COVID-19-related stressors were likely to compound the impacts of preexisting social marginalization, discrimination and abuse.

## Materials and methods

We conducted an online cross-sectional survey of trans Australians utilizing a non-probability snowball sampling approach. The survey was open to Australian residents ≥16 years of age who identified as trans between 1st May 2020 and 30th June 2020. The survey was designed collaboratively by our core team of researchers who are members of the Australian trans community (SZ, AWFQ, AG, TC, KE, ED), with support from clinicians experienced in trans healthcare (LMA, ASC). Survey data were collected and managed using REDCap electronic data capture tools hosted at The University of Melbourne. The study received ethical and governance approval by the Austin Health Human Research Ethics Committee (Reference Number HREC/57155/Austin-2019), ACON Research Ethics Review Committee (Reference Number 2020/03), and the Thorne Harbour Health Community Research Endorsement Panel (Reference Number THH/CREP 20-006).

The survey preamble outlined that completing the survey implied consent. Inclusion criteria were assessed *via* three screening questions: (a) currently living in Australia; (b) identification as trans (“is your gender different to what was presumed for you at birth?”); and (c) aged 16 years or older. Participants were asked to first complete an enrollment survey (as part of a larger longitudinal project) with demographic questions. An individualized link to the “COVID-19 survey” was subsequently sent by email. Duplicate responses or incomplete responses were excluded. All survey questions were optional. Reimbursement (AUD$5 gift card) was provided to participants following completion of the survey.

The survey was posted on social media (Facebook and Instagram). Furthermore, over 100 trans community support groups and organizations in Australia were directly contacted to share the survey within their networks.

Demographic data, including state of residence, age, presumed gender at birth, and gender identity were ascertained. To facilitate data analysis, participants were then asked to self-select the most appropriate gender category of three: trans man, trans woman, or non-binary (see Table 1).

**Table 1.** Demographic characteristics of the study sample.

Demographic variable	*N*	%	National data (%)
*Age (N = 1017)*			
16–25	368	36.2	12.8
26–35	344	33.9	14.4
36–45	121	12.0	13.5
46–55	95	9.4	13.3
56 − 65	61	6.0	11.8
66–75	24	2.4	8.9
76–85	3	0.3	4.8
*Sex presumed at birth (N = 1019)*			
Male	469	46.0	49.1
Female	532	52.2	50.9
Unsure/prefer not to say	18	1.8	NA
*Variation of sex characteristics (Intersex) (N = 1019)*			
No	827	81.2	NA
Yes	88	8.6	NA
Unknown	100	9.8	NA
Prefer not to say	4	0.4	NA
*Gender category (N = 1019)*			
Trans woman	396	38.9	NA
Trans man	362	35.5	NA
Non-binary	261	25.6	NA
*Aboriginal or Torres Strait Islander (N = 1019)*			
Aboriginal	89	8.7	3.0
Torres Strait Islander	21	2.1	0.2
Both Aboriginal and Torres Strait Islander	12	1.2	0.1
Non-Indigenous	885	86.9	96.7
Prefer not to say	12	1.2	NA
*Country of birth (N = 1019)*			
Australia	859	84.3	70.3
Other	160	15.7	29.7
*State/territory of residence (N = 1019)*			
Australian Capital Territory	53	5.2	1.7
New South Wales	255	25.0	32.1
Northern Territory	11	1.1	0.9
South Australia	132	13.0	7.0
Queensland	66	6.5	19.8
Tasmania	25	2.5	2.1
Victoria	375	36.8	26.2
Western Australia	102	10.0	10.2

NA equals not applicable. Source of National Data: Australian Bureau of Statistics 2020, and Australian Institute of Health and Welfare 2019 Profile of Indigenous Australians.

Participants were asked “Has your living situation changed in response to COVID-19?” with Yes or No response options; as well as “What statement best describes your current living situation at the moment?” with fixed-response and open-ended options, as outlined in Table 2. To explore safety during social isolation, participants were asked “Does anyone in your household make you feel unsafe or afraid?” with Yes or No response options. Changes in employment as a result of the COVID-19 pandemic were assessed with fixed-responses to “What best describes your current employment status?” and “How has your employment status changed because of the COVID-19 pandemic?” specifically with the option to select all that applied from (a) I lost my job; (b) I am working reduced hours; (c) Contact with work colleagues reduced; (d) I was unemployed prior to the COVID-19 pandemic; (e) it has not been affected; and (f) other. For the purposes of statistical analysis, “job loss” was categorized as any participant who selected “I lost my job”.

**Table 2.** Living situation, employment, and finances.

Living situation, employment and financial variable	*N*	%	National data (%) (Fisher et al., 2020; Morgan et al., 2020)
*Change of living situation during COVID-19 (N = 1013)*			
Yes	273	27.0	NA
No	740	73.1	NA
*Current household composition (N = 1017)*			
With family (logical or chosen)	300	29.5	NA
With partner(s)	288	28.3	NA
Alone	187	18.4	19.2
With friends or housemates	185	18.2	6.9
With some but not all partners	30	3.0	NA
Mixed household (e.g. parent and housemates)	11	1.1	NA
No regular place of residence	4	0.4	NA
Assisted living/care facility	4	0.4	NA
Boarding school or residential college	4	0.4	NA
Shared custody arrangement	3	0.3	NA
Foster care	1	0.1	NA
*Feeling unsafe or afraid in household (N = 1016)*			
Yes	119	11.7	11.6
No	897	88.3	88.4
*Change in employment status due to COVID-19 (N = 909)*			
No change in employment	289	31.8	NA
Contact with work colleagues greatly reduced	217	23.9	NA
Working reduced hours	200	22.0	NA
Unemployed prior to the pandemic	142	15.6	NA
Lost employment	106	11.7	11.2
Other (e.g. increase in work hours)	146	16.1	NA
*Current employment status (N = 1015)*			
Full time employment	309	30.4	NA
Student	237	23.4	NA
Unemployment	227	22.4	7·4
Part time employment	185	18.2	NA
Casual employment	156	15.4	NA
Pension	97	9.6	NA
Volunteer	50	4.9	NA
House duties	44	4.3	NA
Retired	21	2.1	NA
*COVID-19 related financial strain (N = 1019)*			
Rent/mortgage	240	23.6	NA
Utilities (e.g. electricity, gas, water, internet)	260	25.5	NA
Food/groceries	318	31.2	NA
Financially supporting others	145	14.2	NA
Other (e.g. medication, healthcare)	106	10.4	NA

NA – not applicable. National data are for the same time period. Source of National Data: Fisher et al. (2020) and Morgan et al. (2020). Canberra: Australian Institute of Criminology, 2020, and Employment, hours worked and unemployment rise in June. Australian Bureau of Statistics 2020.

Participants were asked “Has the COVID-19 pandemic put financial strain on any of the following?” with the option to select all that applied from; (a) rent/mortgage; (b) utilities (e.g. electricity, gas, water, internet); (c) food/groceries; (d) provision of financial support to others; (e) other (open-text response). For purposes of analysis, “financial strain” was categorized as any participant who had indicated one or more of the forms of financial strain.

Depression and thoughts of self-harm or suicide were assessed using the PHQ-9 (Arroll et al., 2010; Kroenke et al., 2001). The PHQ-9 was chosen given the availability of Australian normative data (Fisher et al., 2020), and validation against formal diagnostic psychiatric interviews (Arroll et al., 2010; Staples et al., 2019). PHQ-9 is an easy to understand, self-reported 9-item scale, whereby respondents select the severity of nine depressive symptoms as “0” (not experienced) to “3” (experienced nearly every day). The sum of all nine responses provide a total score. PHQ-9 scores ≥10 are 88% sensitive and 85% specific for detecting clinically significant major depression (Levis et al., 2019). PHQ-9 scores of 5–9 represent mild, 10–14 moderate, 15–19 moderately severe, and ≥20 severe depressive symptoms. Specifically, PHQ-9 Item 9 assessed thoughts of self-harm or suicide (“thoughts that you would be better off dead or of hurting yourself in some way”).

Descriptive frequencies were reported, and median (interquartile range) values were included for not normally distributed data. Statistical analysis was performed using R version 4.0.2 (R Foundation for Statistical Computing, Vienna, Austria). Logistic regression was performed to explore associations between experiences of COVID-19-related stressors and depression and thoughts of self-harm or suicide. Models for depression and responses to item-9 (thoughts that one would be better off dead or of hurting themselves) were analyzed separately with four types of experiences of COVID-19 – job loss (participants who indicated “I lost my job”), feeling unsafe or afraid in household, financial strain (participants who indicated financial strain in relation to at least one of housing, utilities, groceries, financial supporting others, or “other”), and surgery canceled or postponed. All models were adjusted for age, being born overseas, gender category, and living situation to allow for similar comparisons with a national survey (Fisher et al., 2020).

## Results

A total of 1162 responses were received. After removing duplicates, ineligible responses and incomplete surveys, 1019 participants remained.

### Demographic data

Demographic data are summarized in Table 1. The median age of participants was 29 years (range 16–80). Responses were received from participants living in all Australian states and territories, though were not represented proportionately to the population. There was a greater number of younger individuals, and a higher proportion of First Nations Aboriginal or Torres Strait Islander people in our sample than national averages (Table 1). The proportion of individuals identifying as trans women, trans men, and non-binary in this sample were similar to another trans adult community survey in Australia (Zwickl et al., 2019).

### Living situation

Since the onset of the COVID-19 pandemic, 27% (*n* = 273) of participants reported that their living situation had changed. Reasons for a change in living situation included job loss, financial strain, and attempts to ensure ongoing access to informal supports during social restrictions by combining formerly separate households. Household composition is outlined in Table 2. A total of 11.7% reported that they were living with someone that made them feel unsafe or afraid, which is comparable to Australian general population reports of 11.6% during the early stages of the COVID-19 pandemic (Morgan et al., 2020).

### Employment and financial situation

The majority of the participants experienced some negative change in employment status as outlined in Table 2. Over a third had reduced working hours or had become unemployed. Approximately, one in four experienced social impacts, such as reduction in contact with work colleagues. Almost half of participants (*n* = 550) reported experiencing financial strain related to the COVID-19 pandemic.

### Patient health questionnaire-9 (PHQ-9)

The PHQ-9 was completed by 985 participants (Table 3). Of note, 61.1% (*n* = 602) of participants experienced clinically significant symptoms of depression (PHQ-9 score ≥10). This is significantly higher than 27.6% reported in the general Australian population in response to COVID-19 during May 2020 (Fisher et al., 2020), and higher than in trans Australians prior to the pandemic (36% and 44% reported to have PHQ-9 score ≥10 in 2009 by Pitts et al. and in 2013 by Hyde et al. respectively). In a subgroup analysis by gender (trans men, trans women, or non-binary shown in Table 3), the non-binary group was more likely to experience clinically significant symptoms of depression compared to binary groups (74.3% in non-binary group compared to 59.9% in trans men and 53.8% in trans women, all overall *p* values <0.01).

**Table 3.** Depression and thoughts of self-harm or suicide (PHQ-9).

Mental health variable	Trans men *N* (%)	Trans women *N* (%)	Non-binary *N* (%)	Total *N* (% )	National data (%) (Fisher et al., 2020)
PHQ-9 score and depression severity (N = 985)*					
0–4 (minimal or none)	46 (13.3)	72 (18.9)	22 (8.6)	140 (14.2)	NA
5–9 (mild)	95 (27.4)	104 (27.3)	44 (17.1)	243 (24.7)	26.5
10–14 (moderate)	77 (22.2)	77 (20.2)	64 (24.9)	218 (22.1)	A total of 27.6 (score ≥ 10)
15–19 (moderately severe)	66 (19.0)	66 (17.3)	57 (22.2)	189 (19.2)	
20–27 (severe)	65 (18.7)	62 (16.3)	70 (27.2)	195 (19.8)	
PHQ-9 – item 9 Thoughts that you would be better off dead or of hurting yourself in some way (last two weeks) (N = 985)**					
Not at all	189 (54.5)	205 (53.8)	108 (42.0)	502 (51.0)	85.4
Several days	73 (21.0)	91 (23.9)	71 (27.6)	235 (23.9)	8.9
More than half the days	51 (14.7)	49 (12.9)	31 (12.1)	131 (13.3)	3.0
Nearly every day	34 (9.8)	36 (9.5)	47 (18.3)	117 (11.9)	2.7

NA equals not applicable. Source of National Data: Fisher et al. (2020).

* Overall *p* value from Chi-squared test comparing between non-binary and binary (trans men *p* = 0.004 and trans women *p* < 0.0001).

** Overall *p* value from Chi-squared test comparing between non-binary and binary (trans men *p* = 0.001 and trans women *p* = 0.002).

Notably, 49% (*n* = 483) of participants reported that they had thought that they would be better off dead or of hurting themselves in the two preceding weeks, which is almost double the rate reported by Pitts et al. (2009), though similar to Hyde et al. (2013). A total of 11.9% (*n* = 117) reported that they experienced these thoughts nearly every day. The occurrence of such thoughts during the COVID-19 pandemic in 49% of trans Australian adults was significantly higher than 14.9% of the general Australian population (Fisher et al., 2020). Individuals with non-binary identities reported a higher prevalence of having thoughts that they would be better off dead or of hurting themselves in the prior two weeks compared to individuals with binary identities (Table 3). A descriptive table of the PHQ-9 and Item 9 scores by state and territory has been included as a Supplementary Table.

Predictors of clinically significant depression or a participant selecting that they had experienced “thoughts that you would be better off dead or of hurting yourself in some way” are outlined in Table 4. Contrary to national data, job loss due to COVID-19 restrictions was not statistically associated with a higher risk of depression or thoughts that they would be better off dead or of hurting themselves in Australian trans individuals. Notably, the unemployment rate was 22.4% which is three times higher than the national rate (Table 2). Feeling unsafe or afraid in the household and financial strain posed a higher risk for both depression and having thoughts that they would be better off dead or of hurting themselves. Cancelation or postponement of gender-affirming surgery due to COVID-19 was associated with a 56% increase in the risk of having thoughts that they would be better off dead or of hurting themselves (Table 4).

**Table 4.** Associations between experiences of COVID-19 and depression and thoughts of self-harm or suicide.

	Mental health outcome (last two weeks)			
	Clinically significant symptoms of depression (PHQ9 score >10)		Thoughts that you would be better off dead or of hurting yourself in some way	
(*N* = 985)	Trans sample OR (95% CI)*	National data (Fisher et al., 2020)	Trans Sample OR (95% CI)*	National data (Fisher et al., 2020)
Job loss due to COVID-19 restrictions	0.70 (0.44, 1.11)	**1**.**50 (1**.**31, 1**.**72)**	1.11 (0.71, 1.73)	**1**.**31 (1**.**11, 1**.**55)**
Feeling unsafe or afraid in household	**1**.**75 (1**.**06, 2**.**89)**	NA	**1**.**96 (1**.**23, 3**.**08)**	NA
Financial strain	**1**.**85 (1**.**69, 2.47)**	NA	**1**.**80 (1**.**36, 2**.**38)**	NA
Gender-affirming surgery canceled or postponed	1.35 (0.88, 2.07)	NA	**1**.**56 (1**.**04, 2**.**35)**	NA

NA equals not applicable. Bold values indicate odds ratios where its corresponding 95% confidence interval does not cross 1.

* Odds ratio (95% CI) for all four types of experiences of COVID-19 are mutually adjusted for each other with age, being born overseas, gender, and living situation also included as covariates. National data from Fisher et al. (2022).

## Discussion

This large community survey involving 1019 participants is one of the first studies describing the impact of the COVID-19 pandemic on the trans community in Australia. These data quantify the magnitude and severity of depression and thoughts of self-harm or suicide in the first three months of the COVID-19 pandemic. Concerningly, 61% of trans Australians met criteria based on PHQ-9 for clinically significant depression, considerably higher than prior to the pandemic (rates of 36% reported in 2009 by Pitts et al. and 44% in 2013 by Hyde et al.) and more than twice the rate seen in the Australian general population during the pandemic (Fisher et al., 2020). Additionally, almost half the participants (49%) reported thoughts of self-harm or suicide in the preceding two weeks, which was significantly more likely in people who reported feeling unsafe or afraid in their household, experienced financial strain, or had cancelation or postponement of planned gender-affirming surgery. Rates of experiencing thoughts of self-harm or suicide are higher than the general Australian population (Fisher et al., 2020) but are similar to previous reports in trans Australians (Hyde et al., 2013).

Trans Australians with non-binary identities reported higher rates of both depression and thoughts of self-harm or suicide compared to those with binary identities which is consistent with findings from prior to the pandemic (Cheung et al., 2020; James et al., 2016). This may be related to a lack of social and legal recognition of non-binary genders (McLemore, 2015; Valentine, 2016) and is unlikely to be attributed to the COVID-19 pandemic.

The overall high rates of clinically significant depression and thoughts of self-harm or suicide are likely the result of the preexisting effects of social marginalization, discrimination, and high rates of physical and verbal abuse and associated high rates of depression and suicidality experienced by the trans community (Bretherton et al., 2021; Strauss et al., 2020), *compounded by* COVID-19 pandemic-related stressors. In addition to ongoing challenges faced by the trans community, trans Australians may have faced isolation from trans community and wider support networks and some have experienced disruptions to their gender-affirming healthcare through cancelation of surgeries.

Feeling unsafe or afraid in the household posed a higher risk for both depression and thoughts of self-harm or suicide. Rates of feelings unsafe or unafraid in the household were comparable with the general population (Fisher et al., 2020), and therefore it cannot be presumed that such experiences are related to one’s trans status. There is, however, some evidence that many trans people face discrimination and violence within the home (James et al., 2016; Riggs et al., 2015; Smith et al., 2014) and that this is associated with poorer mental health (Riggs et al., 2015).

There were significantly greater odds of thoughts of self-harm or suicide in trans people experiencing cancelation or postponement of their gender-affirming surgery. Gender-affirming surgery can be a critical part of transition and affirmation for many trans people, with previous data demonstrating that access to gender-affirming surgery is protective against suicidal ideation and suicide risk (Bauer et al., 2015; Tucker et al., 2018). Despite the 95% confidence interval crossing 1 for reporting depression, the point estimate indicates an increased odds ratio of 1.35 for cancelation of surgery. Statistically, whilst the concordance rate between depression and suicide is high (76%), there is a group of individuals (18%) who met criteria for clinically significant depression but did not have thoughts of self-harm or suicide. Amongst those who had thoughts of self-harm or suicide and had surgery canceled, the majority (93%) also had depression. In contrast, amongst those who had surgery canceled and no thoughts of self-harm or suicide, only 29% had depression. This may suggest that cancelation of surgery may not be a primary risk factor for some individuals with depression but no thoughts of self-harm or suicide. This is likely contributing to the (relatively) smaller effect size between surgery canceled and depression, in contrast to the effect size for thoughts of self-harm or suicide. With resumption of elective surgery, prioritization of gender-affirming surgery may help alleviate symptoms of depression and thoughts of self-harm or suicide in the trans community which are clearly higher than the general population.

The increased financial strain resulting from the COVID-19 pandemic is associated with 80% higher odds of experiencing depression or thoughts of self-harm or suicide, which disproportionately impacted an already economically marginalized community. Contrary to general population data, job loss itself during the COVID-19 pandemic was not statistically associated with a higher risk of depression or thoughts of self-harm or suicide in trans Australians. Notably, the national data (Fisher et al., 2020) was collected in the first month of COVID-19 restrictions in Australia (3rd April–2nd May 2020) and found an increased odds of reporting depression or thoughts of self-harm/suicide with job loss at a time prior to any tangible government assistance. This survey was conducted between the 1st May and 30th June 2020. From the first week of May 2020, the Australian Government began paying businesses who were adversely affected by the COVID-19 pandemic a wage subsidy (known as JobKeeper) to enable them to keep employees in jobs. This flat payment of AUD$1500 per fortnight was the equivalent of 70% of the national median wage. This likely provided financial relief and job certainty for at least six months for many individuals and for some, JobKeeper payments were higher than their usual income, providing positive financial benefits. This complexity likely explained the lack of associations with job loss in our survey. There are also potential confounding effects with financial strain, in which despite evidence of an association in univariate analysis, there is a considerable change in the estimated odds ratio of job loss (>10%) when financial strain was included in the model.

With ongoing uncertainty surrounding the pandemic and intermittent implementation of social restrictions, there is likely to be ongoing issues of unemployment, financial strain, and unsafe living situations, coupled with fear and social isolation. These are all likely to have a long-term adverse impact on mental health. Suicidality is likely to present for longer and peak later than the pandemic (Gunnell et al., 2020), with great fears for a suicide epidemic in the trans community (Wang et al., 2020).

Overall, mental health services and support are critical to addressing the high rates of depression and thoughts of self-harm or suicide in the Australian trans community during and after the COVID-19 pandemic. Whilst Australia’s mental health sector has been agile in responding to the needs exposed by the COVID-19 pandemic, including expanding telehealth, mainstream services are often inept in their understanding of the trans experience and therefore the complex mental health needs of many trans people (Strauss et al., 2020; Zwickl et al., 2019). Certainly, previous research has found that LGBTIQA + individuals avoid mainstream telephone crisis counseling because they anticipate discrimination (Waling et al., 2020). Given the unique and complex challenges that trans people often face, mainstream mental health services should be provided with additional trans competency training, and specialized LGBTIQA + and trans-specific services require additional funding and resources to increase their capacity to meet the increase in demand. Safe and affirming mental health support strategies that can be delivered safely within COVID-19 social restrictions need to be explored, and potentially online-based peer support programs, smartphone-based applications, or text messaging may be useful options. The financial accessibility of mental health support should also be considered, given the high rates of unemployment and financial strain experienced by the trans community. In addition, given that loss of employment and financial strain are well-recognized risk factors for suicide in the general population (Blakely et al., 2003; Classen & Dunn, 2012; Nordt et al., 2015), both issues require urgent government attention.

### Limitations

There are multiple limitations to this cross-sectional study and based upon a non-probability snowball sampling approach. This study identified associations but not causal relationships. The online-based recruitment may explain why a greater proportion of responders were younger individuals and hence may not accurately reflect the views of the older trans community, those who are less computer proficient or in people who may have difficulty with English fluency. Not all areas of Australia were represented equally, as recruitment was not targeted. However, the predominance of respondents in south eastern states is in line with previous Australian trans community surveys (Bretherton et al., 2021; Strauss et al., 2020). The lack of an objective measure of anxiety is also a significant limitation of this study, given that there has been a noted increase in anxiety in the general population during the pandemic (Fisher et al., 2020). Additionally, the survey did not clarify whether feeling unsafe or afraid in one’s household was related to being trans.

Nonetheless, this survey provided a platform for participants to express their views at a time when in-person interviews are not feasible during COVID-19 social restrictions. This is one of few studies describing the impact of the COVID-19 pandemic on trans people who are traditionally marginalized and underrepresented in research. Our use of the standardized PHQ-9 additionally allows comparisons with the general population during COVID-19 social restrictions and outside of COVID-19.

## Conclusion

An urgent, targeted public health response co-created with trans individuals is needed to address the alarming rates of depression and thoughts of self-harm and suicide in trans Australians. COVID-19 pandemic-related stressors appear to have further exacerbated preexisting high rates of depression. Strategies to ensure the safety of trans people to live without discrimination, abuse or violence are needed, particularly in home environments during social restrictions. Moreover, our findings highlight the importance of gender-affirming surgery for trans people and reinstating access may aid in preventing suicide.

## Supplementary Material

Supplemental Material

## Data Availability

Deidentified participate data are available upon reasonable request from the corresponding author via email (adac@unimelb.edu.au), provided that the related research is deemed to be of benefit to the trans and gender diverse community and has undergone Austin Health Human Research Ethics Committee approval in the form of an amendment.

## References

[CIT0001] Adams, N., Hitomi, M., & Moody, C. (2017). Varied reports of adult transgender suicidality: Synthesizing and describing the peer-reviewed and gray literature. *Transgender Health*, *2*(1), 60–75. 10.1089/trgh.2016.0036 28861548 PMC5436370

[CIT0002] Åhs, J. W., Dhejne, C., Magnusson, C., Dal, H., Lundin, A., Arver, S., Dalman, C., & Kosidou, K. (2018). Proportion of adults in the general population of Stockholm County who want gender-affirming medical treatment. *PloS One*, *13*(10), e0204606. 10.1371/journal.pone.0204606 30289896 PMC6173394

[CIT0003] Arroll, B., Goodyear-Smith, F., Crengle, S., Gunn, J., Kerse, N., Fishman, T., Falloon, K., & Hatcher, S. (2010). Validation of PHQ-2 and PHQ-9 to screen for major depression in the primary care population. *Annals of Family Medicine*, *8*(4), 348–353. 10.1370/afm.1139 20644190 PMC2906530

[CIT0004] Bauer, G. R., Scheim, A. I., Pyne, J., Travers, R., & Hammond, R. (2015). Intervenable factors associated with suicide risk in transgender persons: A respondent driven sampling study in Ontario, Canada. *BMC Public Health*, *15*(1), 1–15. 10.1186/s12889-015-1867-2 26032733 PMC4450977

[CIT0005] Blakely, T. A., Collings, S. C. D., & Atkinson, J. (2003). Unemployment and suicide. Evidence for a causal association? *Journal of Epidemiology and Community Health*, *57*(8), 594–600. 10.1136/jech.57.8.594 12883065 PMC1732539

[CIT0006] Bockting, W. O., Miner, M. H., Swinburne Romine, R. E., Hamilton, A., & Coleman, E. (2013). Stigma, mental health, and resilience in an online sample of the US transgender population. *American Journal of Public Health*, *103*(5), 943–951. 10.2105/AJPH.2013.301241 23488522 PMC3698807

[CIT0007] Bradbury-Jones, C., & Isham, L. (2020). The pandemic paradox: The consequences of COVID-19 on domestic violence. *Journal of Clinical Nursing*, *29*(13–14), 2047–2049. 10.1111/jocn.15296 32281158 PMC7262164

[CIT0008] Bretherton, I., Thrower, E., Zwickl, S., Wong, A., Chetcuti, D., Grossmann, M., Zajac, J. D., & Cheung, A. S. (2021). The health and well-being of transgender Australians: A national community survey. *LGBT Health*, *8*(1), 42–49. 10.1089/lgbt.2020.0178 33297824 PMC7826417

[CIT0009] Cheung, A. S., Leemaqz, S. Y., Wong, J. W. P., Chew, D., Ooi, O., Cundill, P., Silberstein, N., Locke, P., Zwickl, S., Grayson, R., Zajac, J. D., & Pang, K. C. (2020). Non-binary and binary gender identity in Australian trans and gender diverse individuals. *Archives of Sexual Behavior*, *49*(7), 2673–2681. 10.1007/s10508-020-01689-9 32285311

[CIT0010] Classen, T. J., & Dunn, R. A. (2012). The effect of job loss and unemployment duration on suicide risk in the United States: A new look using mass-layoffs and unemployment duration . *Health Economics*, *21*(3), 338–350. 10.1002/hec.1719 21322087 PMC3423193

[CIT0011] Crissman, H. P., Berger, M. B., Graham, L. F., & Dalton, V. K. (2017). Transgender demographics: A household probability sample of US adults, 2014. *American Journal of Public Health*, *107*(2), 213–215. 10.2105/AJPH.2016.303571 27997239 PMC5227939

[CIT0012] Fisher, J. R., Tran, T. D., Hammarberg, K., Sastry, J., Nguyen, H., Rowe, H., Popplestone, S., Stocker, R., Stubber, C., & Kirkman, M. (2020). Mental health of people in Australia in the first month of COVID-19 restrictions: A national survey. *The Medical Journal of Australia*, *213*(10), 458–464. 10.5694/mja2.50831 33107063 PMC7756394

[CIT0013] Gunnell, D., Appleby, L., Arensman, E., Hawton, K., John, A., Kapur, N., Khan, M., O’Connor, R. C., & Pirkis, J., (2020). Suicide risk and prevention during the COVID-19 pandemic. *The Lancet. Psychiatry*, *7*(6), 468–471. 10.1016/S2215-0366(20)30171-1 32330430 PMC7173821

[CIT0014] Hyde, Z., Doherty, M., Tilley, M., McCaul, K., Rooney, R., Jancey, J. (2013). *The first Australian national trans mental health study: Summary of results*. Retrieved from https://www.beyondblue.org.au/docs/default-source/research-project-files/bw0288_the-first-australian-national-trans-mental-health-study–-summary-of-results.pdf?sfvrsn=2

[CIT0015] Jaffee, K. D., Shires, D. A., & Stroumsa, D. (2016). Discrimination and delayed health care among transgender women and men: Implications for improving medical education and health care delivery. *Medical Care*, *54*(11), 1010–1016. 10.1097/MLR.0000000000000583 27314263

[CIT0016] James, S., Herman, J., Rankin, S., Keisling, M., Mottet, L., Anafi, M. A. (2016). *The report of the 2015 US transgender survey*. Washington, DC. Retrieved from https://transequality.org/sites/default/files/docs/usts/USTS-Full-Report-Dec17.pdf

[CIT0017] Kiely, K. M., & Butterworth, P. (2015). Validation of four measures of mental health against depression and generalized anxiety in a community based sample. *Psychiatry Research*, *225*(3), 291–298. 10.1016/j.psychres.2014.12.023 25578983

[CIT0018] Kroenke, K., Spitzer, R. L., & Williams, J. B. (2001). The PHQ-9: Validity of a brief depression severity measure. *Journal of General Internal Medicine*, *16*(9), 606–613. 10.1046/j.1525-1497.2001.016009606.x 11556941 PMC1495268

[CIT0019] Lai, M.-C., Chiu, Y.-N., Gadow, K. D., Gau, S. S.-F., & Hwu, H.-G. (2010). Correlates of gender dysphoria in Taiwanese university students. *Archives of Sexual Behavior*, *39*(6), 1415–1428. 10.1007/s10508-009-9570-y 19937374

[CIT0020] Levis, B., Benedetti, A., & Thombs, B. D. (2019). Accuracy of patient health questionnaire-9 (PHQ-9) for screening to detect major depression: Individual participant data meta-analysis. *BMJ*, *365*, l1476. 10.1136/bmj.l1476 30967483 PMC6454318

[CIT0021] McLemore, K. A. (2015). Experiences with misgendering: Identity misclassification of transgender spectrum individuals. *Self and Identity*, *14*(1), 51–74. 10.1080/15298868.2014.950691

[CIT0022] Moody, C., & Smith, N. G. (2013). Suicide protective factors among trans adults. *Archives of Sexual Behavior*, *42*(5), 739–752. 10.1007/s10508-013-0099-8 23613139 PMC3722435

[CIT0023] Morgan, A., Boxall, H., Brown, R. (2020). *The prevalence of domestic violence among women during the COVID-19 pandemic: Technical appendix*. Australian Government. Retrieved from https://www.aic.gov.au/sites/default/ files/2020-08/sb28_technical_appendix-revised-prevalence_of_domestic_violence_among_women_ during_covid-19_pandemic.pdf

[CIT0024] Nordt, C., Warnke, I., Seifritz, E., & Kawohl, W. (2015). Modelling suicide and unemployment: A longitudinal analysis covering 63 countries, 2000–11. *The Lancet. Psychiatry*, *2*(3), 239–245. 10.1016/S2215-0366(14)00118-7 26359902

[CIT0025] Pierce, M., Hope, H., Ford, T., Hatch, S., Hotopf, M., John, A., Kontopantelis, E., Webb, R., Wessely, S., McManus, S., & Abel, K. M. (2020). Mental health before and during the COVID-19 pandemic: A longitudinal probability sample survey of the UK population. *The Lancet. Psychiatry*, *7*(10), 883–892. 10.1016/S2215-0366(20)30308-4 32707037 PMC7373389

[CIT0026] Pitts, M. K., Couch, M., Mulcare, H., Croy, S., & Mitchell, A. (2009). Transgender people in Australia and New Zealand: Health, well-being and access to health services. *Feminism & Psychology*, *19*(4), 475–495. 10.1177/0959353509342771

[CIT0027] Riggs, D. W., Ansara, G. Y., & Treharne, G. J. (2015). An evidence-based model for understanding the mental health experiences of transgender Australians. *Australian Psychologist*, *50*(1), 32–39. 10.1111/ap.12088

[CIT0028] Riggs, D. W., Coleman, K., & Due, C. (2014). Healthcare experiences of gender diverse Australians: A mixed-methods, self-report survey. *BMC Public Health*, *14*(1), 230. 10.1186/1471-2458-14-230 24597614 PMC3973980

[CIT0029] Riggs, D. W., von Doussa, H., & Power, J. (2015). The family and romantic relationships of trans and gender diverse Australians: An exploratory survey. *Sexual and Relationship Therapy*, *30*(2), 243–255. 10.1080/14681994.2014.992409

[CIT0030] Sherman, A. D., Clark, K. D., Robinson, K., Noorani, T., & Poteat, T. (2020). Trans* community connection, health, and wellbeing: a systematic review. *LGBT Health*, *7*(1), 1–14. 10.1089/lgbt.2019.0014 31794289

[CIT0031] Smith, E., Jones, T., Ward, R., Dixon, J., Mitchell, A., Hillier, L. (2014). *From blues to rainbows: Mental health and wellbeing of gender diverse and transgender young people in Australia*. Retrieved from Melbourne: https://www.beyondblue.org.au/docs/default-source/research-project-files/bw0268-from-blues-to-rainbows-report-final-report.pdf?sfvrsn=2

[CIT0032] Staples, L. G., Dear, B. F., Gandy, M., Fogliati, V., Fogliati, R., Karin, E., Nielssen, O., & Titov, N. (2019). Psychometric properties and clinical utility of brief measures of depression, anxiety, and general distress: The PHQ-2, GAD-2, and K-6. *General Hospital Psychiatry*, *56*, 13–18. 10.1016/j.genhosppsych.2018.11.003 30508772

[CIT0033] Strauss, P., Cook, A., Winter, S., Watson, V., Wright Toussaint, D., & Lin, A. (2020). Associations between negative life experiences and the mental health of trans and gender diverse young people in Australia: Findings from Trans Pathways. *Psychological Medicine*, *50*(5), 808–817. 10.1017/S0033291719000643 31280740

[CIT0034] Strauss, P., Lin, A., Winter, S., Waters, Z., Watson, V., Wright Toussaint, D., & Cook, A. (2020). Options and realities for trans and gender diverse young people receiving care in Australia’s mental health system: Findings from Trans Pathways. *Australian & New Zealand Journal of Psychiatry*. 10.1177/0004867420972766 33198483

[CIT0035] Tucker, R. P., Testa, R. J., Simpson, T. L., Shipherd, J. C., Blosnich, J. R., & Lehavot, K. (2018). Hormone therapy, gender affirmation surgery, and their association with recent suicidal ideation and depression symptoms in transgender veterans. *Psychological Medicine*, *48*(14), 2329–2336. 10.1017/S0033291717003853 29331161

[CIT0036] Valentine, V. (2016). *Non-binary people’s experiences in the UK*. Retrieved from Edinburgh: https://www.scottishtrans.org/wp-content/uploads/2016/11/Non-binary-report.pdf

[CIT0037] Waling, A., Lim, G., Dhalla, S., Lyons, A., & Bourne, A. (2020). *Understanding LGBTI + Lives in Crisis*. Retrieved from Bundoora, VIC & Canberra, ACT.

[CIT0038] Wang, Y., Pan, B., Liu, Y., Wilson, A., Ou, J., & Chen, R. (2020). Health care and mental health challenges for transgender individuals during the COVID-19 pandemic. *The Lancet. Diabetes & Endocrinology*, *8*(7), 564–565. 10.1016/S2213-8587(20)30182-0 32445629 PMC7239622

[CIT0039] White Hughto, J. M., & Reisner, S. L. (2016). A systematic review of the effects of hormone therapy on psychological functioning and quality of life in transgender individuals. *Transgender Health*, *1*(1), 21–31. 10.1089/trgh.2015.0008 27595141 PMC5010234

[CIT0040] Wiepjes, C. M., den Heijer, M., Bremmer, M. A., Nota, N. M., de Blok, C. J., Coumou, B. J., & Steensma, T. D. (2020). Trends in suicide death risk in transgender people: Results from the Amsterdam Cohort of Gender Dysphoria study (1972–2017). *Acta Psychiatrica Scandinavica*, *141*(6), 486–491. 10.1111/acps.13164 32072611 PMC7317390

[CIT0041] Zwickl, S., Wong, A., Bretherton, I., Rainier, M., Chetcuti, D., Zajac, J. D., & Cheung, A. S. (2019). Health needs of trans and gender diverse adults in Australia: A qualitative analysis of a national community survey. *International Journal of Environmental Research and Public Health*, *16*(24), 5088. 10.3390/ijerph16245088 31847083 PMC6950552

