# Supplementary material for: The impact of the first three months of the COVID-19 pandemic on the Australian trans community
Source: Int J Transgend Health. 2021 Mar 11;24(3):281–91. doi: 10.1080/26895269.2021.1890659 (PMC10373614; doi:10.1080/26895269.2021.1890659)
Supplement: Supplemental Material [file WIJT_A_1890659_SM9772.docx]

**Supplementary Table**

**Comparison of Depression and Thoughts of Self-harm or Suicide (PHQ-9) by Australian State/Territory**

| **Mental Health Variable** | **ACT**  **N(%)** | **NSW**  **N(%)** | **NT**  **N(%)** | **QLD**  **N(%** ) | **SA**  **N(%** ) | **TAS**  **N(%)** | **VIC**  **N(%)** | **WA**  **N(%)** |
| --- | --- | --- | --- | --- | --- | --- | --- | --- |
| PHQ-9 Score and Depression Severity (N=985) |  |  |  |  |  |  |  |  |
| 0 – 4 (Minimal or none) | 6 (12.0) | 41 (16.5) | 4 (33.3) | 16 (12.3) | 9 (14.3) | 4 (16.7) | 51 (14.3) | 9 (9.0) |
| 5 – 9 (Mild) | 14 (28.0) | 55 (22.2) | 1 (8.3) | 36 (27.7) | 16 (25.4) | 6 (25.0) | 97 (27.1) | 18 (18.0) |
| 10 – 14 (Moderate) | 13 (26.0) | 53 (21.4) | 1 (8.3) | 30 (23.1) | 9 (14.3) | 3 (12.5) | 79 (22.1) | 30 (30.0) |
| 15 – 19 (Moderately Severe) | 13 (26.0) | 49 (19.8) | 0 (0.0) | 25 (19.2) | 7 (11.1) | 6 (25.0) | 65 (18.2) | 24 (24.0) |
| 20 – 27 (Severe) | 4 (8%) | 50 (20.2) | 6 (50.0) | 23 (17.7) | 22 (34.9) | 5 (20.8) | 66 (18.4) | 19 (19.0) |
| PHQ-9 – Item 9 Thoughts that you would be better off dead or of hurting yourself in some way (last two weeks) (N=985) |  |  |  |  |  |  |  |  |
| Not at all | 29 (58.0) | 126 (50.8) | 5 (41.7) | 62 (47.7) | 29 (46.0) | 13 (54.2) | 194 (54.2) | 44 (44.0) |
| Several days | 9 (18.0) | 58 (23.4) | 1 (8.3) | 38 (29.2) | 8 (12.7) | 4 (16.7) | 86 (24.0) | 31 (31.0) |
| More than half the days | 9 (18.0) | 32 (12.9) | 3 (25.0) | 12 (9.2) | 10 (15.9) | 5 (20.8) | 46 (12.9) | 14 (14.0) |
| Nearly every day | 3 (6.0) | 32 (12.9) | 3 (25.0) | 18 (13.9) | 16 (25.4) | 2 (8.3) | 32 (8.9) | 11 (11.0) |
